# The Role of ER Stress-Related Phenomena in the Biology of Malignant Peripheral Nerve Sheath Tumors

**DOI:** 10.3390/ijms22179405

**Published:** 2021-08-30

**Authors:** Anna Walczak, Maciej Radek, Ireneusz Majsterek

**Affiliations:** 1 Department of Clinical Chemistry and Biochemistry, Medical University of Lodz, 90-647 Lodz, Poland; anna.walczak@umed.lodz.pl; 2 Department of Neurosurgery and Peripheral Nerve Surgery, Medical University of Lodz, 90-647 Lodz, Poland; maciej.radek@umed.lodz.pl

**Keywords:** MPNST, ER-stress, UPR

## Abstract

Malignant peripheral nerve sheath tumors (MPNST) are rare but one of the most aggressive types of cancer. Currently, there are no effective chemotherapy strategies for these malignancies. The inactivation of the neurofibromatosis type I (NF1) gene, followed by loss of TP53, is an early stage in MPNST carcinogenesis. NF1 is a negative regulator of the Ras proteins family, which are key factors in regulating cell growth, homeostasis and survival. Cell cycle dysregulation induces a stress phenotype, such as proteotoxic stress, metabolic stress, and oxidative stress, which should result in cell death. However, in the case of neoplastic cells, we observe not only the avoidance of apoptosis, but also the impact of stress factors on the treatment effectiveness. This review focuses on the pathomechanisms underlying MPNST cells physiology, and discusses the possible ways to develop a successful treatment based on the molecular background of the disease.

## 1. Introduction

Malignant peripheral nerve sheath tumors (MPNST) are rare but one of the types of cancer with the highest mortality [1]. They are tumors of Schwann cell lineage or neural crest pluripotent cells that can derive from extraneural soft tissue as well as peripheral nerves. The main risk factors for the MPNST development are: existing benign plexiform neurofibromas and previous radiation therapy, but also germline point mutations, as well as large deletions covering the neurofibromatosis type I (NF1) gene together with flanking regions [1,2]. We know that about 50% of MPNST cases arise in the context of Neurofibromatosis Type 1 [3]. Among carriers of NF1 mutations, the risk of having MPNST is about 80% [4]. It is estimated that metastasis is diagnosed in 40–68% of patients during treatment [5].

Moreover, MPNST are being claimed to be one of the most difficult types of tumors to treat. This difficulty is due to their inherent aggressiveness, but the limitations of both diagnostic and therapeutic methods also contribute to the low effectiveness of treatment. Current treatments include surgical resection and radiotherapy of the affected areas. Surgical resection often is the only efficient way of MPNST treatment with the proper margins [6]. Conventional chemotherapy is not effective.

The prognosis for MPNST patients remains very poor. The five-year overall survival is very low, with the rate of 15–66%, five-year event-free survival of 24–53%, and local recurrence (LR) rate of 20% to 85.7% [7]. MPNST is usually associated with poor prognosis because they not only can form metastasis (e.g., in the lungs, lymph nodes, and liver) but also because they are able to withstand the adverse environmental conditions due to various adaptation mechanisms [8,9,10,11,12,13]. These factors can lead to the induction of endoplasmic reticulum (ER) stress.

ER stress is induced when unfolded proteins accumulate in the endoplasmic reticulum [14]. Cancer cells frequently exhibit high levels of ER stress, which is caused by many factors, such as oxidative stress, hypoxia, and nutrient deprivation [15,16]. Moreover, there is evidence confirming the role of oncogenic Ras expression in an increase of ER stress levels [17]. Once triggered, ER stress is followed by a signal transduction pathway induction, known as the unfolded protein response (UPR) [18]. The UPR is initially involved as a protective mechanism to decrease the level of protein accumulation; however, when ER stress levels become insurmountable, cell death is induced [18]. This observation has led to the speculation that agents that further enhance ER stress in vulnerable cancer cells could be developed as anti-cancer therapies [19,20,21].

## 2. MPNST Pathogenesis

MPNST cells harbor complex rearranged genomes. More and more studies suggest that NF1 loss is a significant but not passable factor for MPNST development. As NF1-associated MPNST develops from NF1-nullizygous plexiform neurofibromas (PN), they acquire mutations in other driver genes such as TP53 and cyclin-dependent kinase inhibitor 2A (CDKN2A). Loss of NF1 is also seen in most sporadic MPNSTs, suggesting that NF1 is an important tumor suppressor in all types of MPNST. Genetic alterations of CDKN2A and TP53 are also observed in sporadic and radiation-associated MPNST [22]. Deletion of CDKN2A disrupts two encoded proteins (p16INK4A and p19ARF) and their associated regulatory cascades [23]. The analysis of NF1-associated tumor progression from PN to MPNST found biallelic NF1 mutations in all tumor stages, chromosome 17p (TP53) loss in primary MPNST and metastasis, but lack of CDKN2A deletions or epidermal growth factor receptor (EGFR) amplifications [24]. Other studies on MPNST have shown frequent losses on chromosomes 1p, 9p, 11, 12p, 14q, 18, 22q, X, and Y, with focal gains on chromosomes 7, 8q, and 15q and no pathognomonic features [25]. Increased expression of genes encoding the EGFR, neuregulin-1 (NRG1) coreceptor erbB2, c-Kit, platelet-derived growth factor-α, and c-Met was also observed in MPNST [25]. It was also shown that epidermal growth factor receptor signaling pathways are associated with tumorigenesis in the NF1:TP53 mouse tumor model [26,27].

Another important factor in the genetic background of MPNST is polycomb repressive complex 2 (PRC2). It modifies the chromatin structure by tri-methylation of trimethylation at lysine 27 of histone H2/H3 (H3K27me2/3) and thus impacts gene activity [28]. Somatic mutations in the Polycomb repressive complex 2 (PRC2) components-Embryonic Ectoderm Development (EED) and Suppressor of Zeste 12 Protein Homolog (SUZ12) were reported in NF1-associated and sporadic MPNST [22,29,30]. Lee et al. showed loss-of-function somatic alterations of SUZ12 and EED in 92% of sporadic, 70% of NF1-associated, and 90% of radiotherapy-associated MPNSTs [30]. The SUZ12 loss enhances colony growth of NF1-deficient (but not NF1 wild-type) glioblastoma cells. SUZ12 is located near NF1 in 17q11.2 and is involved in both type 1 and type 2 microdeletions at the NF1 locus [31].

Furthermore, SUZ12 ablation causes a loss of trimethylation at lysine 27 of histone H3 and increased H3K27 acetylation [29]. Microdeletions in that gene are associated with an increased risk of MPNST, and together with the loss of NF1 and one copy of SUZ12 from a 17q11.2 microdeletion ignites transformation to MPNST [22,31]. PRC2 catalyzes trimethylation of H3K27 and multiple studies have found that significant loss of H3K27me3 in MPNST is associated with poor survival [32]. Therefore, H3K27me3 loss or PRC2 mutation may be a useful biomarker to diagnose MPNST [30].

As for genetics and the pathology of MPNST, recurrent genetic mutations have been identified in recent studies, such as loss-of function in NF1, PRC2, TP53, CDKN2A, which may provide new opportunities for therapeutic intervention [33].

## 3. Proteotoxic Stress

Aneuploidy and increased gene copy number may promote tumor growth by affecting the proliferation and survival regulators [34]. These imbalances can also lead to an increased number of misfolded proteins and protein aggregates that disrupt the cellular machinery that regulates protein folding and degradation. High expression of chaperones was observed in murine fibroblast cells, which suggests that aneuploidy impairs the folding capacity of cellular proteins [35,36]. A large number of aneuploid cell lines share a common set of genetic instabilities and expression alterations, which are indicative of proteotoxic stress, a secondary hallmark of cancer [36].

Aneuploidy leads to slower growth in most contexts due to excessive protein production, secondary to extra chromosomes. Moreover, 90% of solid tumors are aneuploidy [37]. On the other hand, polyploidy has been associated with the induction of autophagy and UPR [38].

MPNST, in particular, is known to have a complex karyotype with numerous chromosomal alterations [39]. The cancer cells must adjust their metabolism to maintain an increased proliferative rate despite the aneuploidy [40]. They may be more dependent on chaperone proteins responsible for attenuating proteotoxic stress and similarly hypersensitive to any further induction of proteotoxic stress. It was shown that Nf1–/– cells acquired tolerance to proteotoxic stress [41]. In MPNST driven by NF1 loss, Heat Shock Factor 1 (HSF1) was overexpressed and activated, which was required for increased tumor cell viability [41].

**Figure 1:**
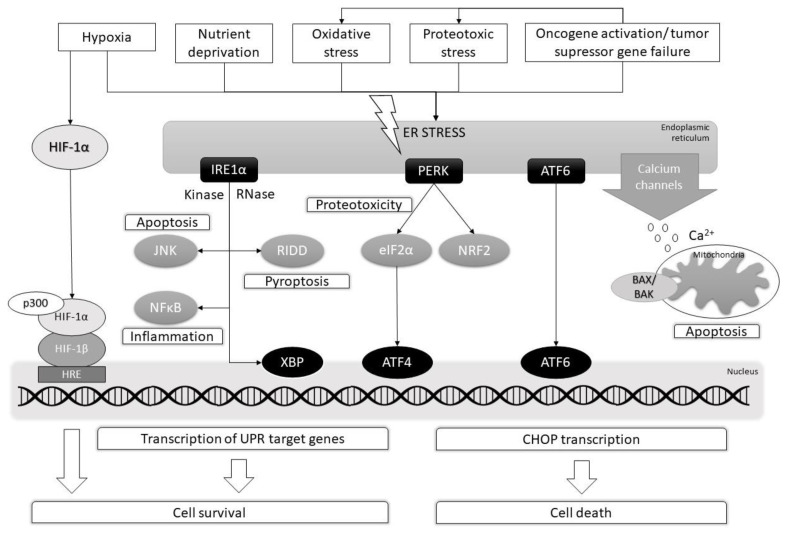
The possible fates of cellular regulation mechanisms under ER stress in MPNST. Various internal and external factors can cause ER stress and concomitant accumulation of unfolded proteins followed by UPR induction. In MPNST cells, ER stress level is elevated (in comparison with normal cells), but at the moderate level, it induces a cascade of IRE1α/PERK/ATF6 signaling pathways to promote cell survival and avoidance of apoptosis. Moreover, HIF-1 is constantly overexpressed in MPNST cells, and its stabilization by hypoxic conditions promotes cell survival and tumor angiogenesis. Currently, one of most interesting therapeutic targets for MPNST are factors that can induce terminal ER stress. Together with Ca^2+^ dysregulation, it can lead to the mitochondrial release of apoptogenic factors that activate caspase-induced apoptosis. Moreover, pro-apoptotic BAX/BAK complex enhances Ca^2+^ release at the ER and mitochondria. When hyperactivated, PERK- and IRE1α-mediated signaling pathways promote inflammation, proteotoxicity as well as pyroptosis and apoptosis. Finally, terminal ER stress signaling induces the transcription of pro-apoptotic factor CHOP.

The unfolded protein response is associated with proteotoxic stress since the ER is the folding site of cellular proteins. Accumulation of unfolded or misfolded proteins in the lumen of the ER leads to the induction of the UPR, which is initially a protective response, but also induces apoptotic cell death in the event of unsolvable ER stress (Figure 1).

Binding immunoglobulin protein (BIP, GRP78) is a luminal ER protein, which induces the UPR. BIP is a chaperone molecule, which can assist in protein folding. When no unfolded proteins are present in the cell, BIP is bound to the three major UPR effectors: Inositol requiring protein 1 (IRE1), Protein kinase RNA-like endoplasmic reticulum kinase (PERK), and activating transcription factor 6 (ATF6), and inhibits their activity.

As the number of unfolded proteins in the ER lumen increases, BIP is recruited away from the UPR initiators to refold these proteins. These proteins are released to trigger the activation of downstream UPR effector proteins. BIP is also responsible for maintaining the permeability barrier of the ER during protein translocation, targeting misfolded proteins for retrograde translocation so they can be degraded by the proteasome, contributing to the formation of calcium stores in the ER and sensing stress conditions in these organelles to activate UPR [42]. MPNSTs cells were shown to be driven by constitutive Ras activation and, therefore, may be subject to substantial ER stress [43]. Raedt et al. (2011) confirmed considerably higher basal levels of ER stress (including BIP overexpression) in MPNST cells compared with normal peripheral nerves [43].

There is some evidence that unfolded proteins can also activate PERK and IRE1 directly, and there is significant crosstalk between the pathways activated by these proteins. PERK is a kinase that oligomerizes under stressful conditions and autophosphorylates itself. It is followed by eukaryotic translation initiation factor 2A (eIF2α) phosphorylation that reduces eIF2α activity, which leads to a suppression of translation, to prevent a further increase in the number of proteins requiring folding in the ER lumen [44].

IRE1α is another transmembrane protein with a luminal stress-sensing domain, with a serine/threonine protein kinase as well as endonuclease activities. Its part containing the cytoplasmic kinase domain may signal to effector pathways to alter protein transcription and translation in response to ER stress [45]. The presence of unfolded proteins causes IRE1α oligomerization and auto-phosphorylation followed by endonucleolytic cleavage of X-box binding protein-1 (XBP-1) mRNA [44]. It was found that both the level of phosphorylated eukaryotic translation initiation factor 2α (eIF2α) and the level of the spliced active form of XBP-1 (sXBP-1) were significantly increased in MPNST cells [43]. The third pathway of the UPR is initiated by ATF6, a transmembrane transcription factor. ATF6 in unstressed cells is retained in the ER lumen by BIP [46]. ER stress causes the release and translocation of ATF6 to the Golgi complex, where it is cleaved by the serine protease site-1 protease (S1P) and the site-2 metalloprotease (S2P) to release a soluble transcription factor that regulates a number of UPR-associated genes [46].

The unfolded protein response is often called a double-edged sword. On the one hand, it triggers the cell-protecting mechanisms that inhibit translation to alleviate stress and promote the degradation of misfolded proteins and aggregates. On the other hand, insurmountable ER stress can also induce UPR-related apoptosis. The enhancement of ER stress with heat shock protein 90 (HSP90) inhibitors coupled with inhibitors of mechanistic target of rapamycin protein (mTOR) led to tumor shrinkage in a genetically engineered murine MPNST model, which correlated with profound ER damage and cell death [43]. In a separate experiment, bortezomib was used as UPR enhancer, and its application reduced MPNST cells viability [47].

The mechanisms causing the switch from an adaptive to a pro-apoptotic response remain unclear. However, thioredoxin interacting protein (TXNIP) may be implicated in the shift to apoptosis [48,49]. In ER stress-activated PERK and IRE1α dependent pathways conditions, TXNIP induces interleukin 1β (IL-1β) mRNA transcription, activates IL-1β production by cryopyrin (NLRP3) inflammasome, and mediates ER stress-mediated β-cell death [50]. Malone et al. (2017) showed mTOR, and HDAC inhibitors kill aggressive malignancies of the nervous system, including MPNST, and cause tumors regression in vivo by converging on the TXNIP/thioredoxin antioxidant pathway through cooperative effects on chromatin and transcription [11]. On the other hand, the suppression of TXNIP promotes cell survival, and also it is an important factor in response to oxidative stress, thus combining the oxidative and proteotoxic pathways [50].

## 4. Hypoxia

Hypoxia is a state in which the level of oxygen in a tissue is not sufficient for the normal functioning of the cells. This phenomenon is common in a majority of malignant tumors due to altered metabolism and uncontrolled cell proliferation. Depriving the tumor of oxygen leads to the induction of vascularization, which is usually dysfunctional, and the acquisition of epithelial-to-mesenchymal transition phenotype contributing to enhanced cell migration. Hypoxia affects cancer cell metabolism, induces genomic and proteomic changes and contributes to therapy resistance by adopting slow-cycling growth (quiescence). It enables the cancer cells to avoid therapies that target rapidly dividing cells and relapse [51]. Hypoxia stimulates a complex cell signaling network in cancer cells, including the hypoxia-inducible factor (HIF), phosphatidylinositol 3-kinase (PI3K), mitogen-activated protein kinase (MAPK) and nuclear factor kappa-light-chain-enhancer of activated B cells (NFĸB) pathways, the crosstalk of which causes positive and negative feedback loops and enhances or reduces the effects of hypoxia [52].

The hypoxia-inducible factor plays a major role in the cellular response to hypoxia. HIF is a heterodimer of an oxygen-labile α subunit and a constitutively expressed β subunit [53]. HIF promotes the expression of more than 150 genes, the products of which are responsible for adaptive responses of cancer cells [51]. Under normoxia, HIF-α protein undergoes rapid proteasomal degradation after ubiquitination. Still, under hypoxic conditions, the degradation process is attenuated and HIF-α localization shifts from the cytoplasm to the nucleus, thus regulating cells metabolism, division and avoidance of apoptosis, tumor cell invasion and metastasis, as well as the expression of pro-angiogenic factors, including vascular endothelial growth factor (VEGF) [54]. The expression of VEGF, downstream of HIF-1α, is also reported to be upregulated in MPNST, suggesting that HIF-1α is responsible for MPNST progression [55]. It was observed that at normal oxygen level, HIF-1α is rapidly degraded, but in MPNST cells, the enhanced expression of HIF-1α in a normoxic environment was also observed [12]. Reports have shown that faster HIF-1α protein synthesis is due to the activation of Akt/mTOR pathway in MPNST and it increases the rate of HIF-1α mRNA [48,56].

HIF-1α usually takes part in adaptive responses under hypoxia to promoting or maintaining tumor cell survival, as confirmed in MPNST cells [12]. The knockdown of HIF-1α in MPNSTs enhanced apoptosis under oxygen deprivation, and the HIF-1α inhibition downregulated downstream genes such as vascular endothelial growth factor A (VEGFA) and glucose transporter 1 GLUT1 [12]. Other MPNST studies also suggested the importance of HIF-1α in MPNST progression in vitro [49]. Rad et al. (2010) demonstrate that cell migration, invasion and tumor formation by signal transducer and activator of transcription 3 (STAT3) are highly dependent on HIF and its knockdown inhibits the prooncogenic activity of STAT3 [49]. Moreover, the inhibition of redox effector factor-1 (Ref-1) and STAT3 impairs the MPNST growth and induces apoptosis [57]. Therefore, HIF-1α seems to be an interesting target in MPNST therapy. 

It was found that chetomin, an inhibitor of HIF-1α/p300 interaction, effectively inhibited the MPNST cell growth and induced apoptosis by attenuating the transcriptional activity of HIF-1α [12]. Moreover, HIF-1α inhibition downregulates the expression of GLUT1 in MPNST cell lines [12]. Another study also suggested a strong association of high GLUT1 expression with inferior outcomes in pediatric patients with MPNST [58]. They also observed overexpression of inflammatory markers (neutrophil-to-lymphocyte ratio, NLR and C-reactive protein-CRP) correlated with expression of carbonic anhydrase 9 (CA9) and GLUT1 in tumors, suggesting a relationship between hypoxia and inflammation in MPNST [58].

## 5. Oxidative Stress

The induction of oxidative stress is a significant event in the cancerous turnover of a normal cell. The generation of reactive oxygen species (ROS) that can interact with DNA causes genetic mutations, induce oncogenes, and promote cancer progression [59]. Thus, it can affect cell proliferation, apoptosis and survival. On the other hand, cancerous cells are also characterized by enhanced ROS production, leading to redox unbalance and an elevated intrinsic oxidative stress. Reactive oxygen species-mediated oncogene activation, such as c-Myc, Bcr-Abl and Ras, as well as TP53-dependent DNA, repair promotion leads to cancer development. Moreover, ROS-induced carcinogenesis is initiated by the activation of several signaling pathways, such as: PI3K/Akt/mTOR, MAPK/ERK, c-Jun N-terminal kinases (JNK), and inactivation of phosphatase and tensin homolog (PTEN) signaling cascades, which in turn modulates the activity of several transcription factors involved in cancer initiation/progression [60]. Moreover, free radicals-related oxidation of prolyl hydroxylase domain protein 2 (PHD2) maintains HIF-1 activity during hypoxia and eases cancer metastasis [61]. The mTOR signaling pathway is constitutively activated in *NF1*-deficient malignancies [62]. Among its various activities, mTOR also regulates the synthesis of reduced glutathione (GSH). mTOR inhibitors in MPNST have been shown to suppress expression levels of the following cellular factors that prevent oxidative damage: sterol regulatory element-binding protein (SREBP), glucose-6-phosphate dehydrogenase (G6PD), and GSH [43].

On the other hand, proto-oncogenes and tumor suppressor genes frequently mutated in cancer commonly cause the accumulation of high amounts of reactive oxygen species [63]. Moreover, tumorigenesis-related phenomena, such as detachment from the extracellular matrix (ECM), hypoxia, and inflammation, can lead to ROS generation, impose further oxidative stress on tumor cells, and induce programmed cell death [63]. On the other hand, some tumor cells adapt to oxidative stress [63]. Transient Receptor Potential Cation Channel A1 (TRPA1) is a neuronal redox-sensing Ca^2+^-influx channel regulating anti-apoptotic signaling pathways [64]. Significant overexpression of TRPA1 mRNA was observed in MPNST in comparison to normal cells [65]. Another study suggests that TRPA1 is upregulated by nuclear factor erythroid 2-related factor 2 (NRF2) and thus induces tolerance to oxidative-stress in tumor cells, including MPNST [66]. Normal cell function depends mainly on interaction with specific components of the extracellular matrix [67]. When they lose the connection with ECM and neighboring cells, they undergo anoikis. Centrally located cells deprived of extracellular matrix have been found to accumulate ROS, which contributes to cell death and the development of a hollow lumen [66]. In the case of MPNST, a decreased level of decorin, an ECM protein, was associated with a poorer prognosis [68]. Another downregulated ECM protein in MPNST is integrin alpha V, a protein responsible for cell adhesion and the inhibition of proliferation [69].

On the other hand, ROS generation may be a useful tool to induce apoptosis in MPNST cells. ROS is known to play an important role in Tumor Necrosis Factor α (TNF α)-mediated cell death and is also implicated in tumor necrosis factor-related apoptosis-inducing ligand (TRAIL) sensitivity [70]. Neurofibromin-deficient MPNST cell lines were sensitive to apoptotic cell death induced by TRAIL, whereas MPNST cells with retained neurofibromin expression or normal human Schwann cells were resistant [71]. The phytochemical curcumin treatment of neurofibromin deficient MPNST cells increased their sensitivity to TRAIL. It was presumably mediated by ROS, as it correlated with increased ROS production, was blocked by N-acetylcysteine and mimicked by exogenous ROS [71]. Another promising therapy was described by Lee et al. (2017). In their study, 5-aminolevulinic mediated photodynamic therapy (photosensitizer precursor used with light to generate reactive oxygen species) combined with doxycycline significantly decreased the viability of MPNST cells [72].

## 6. The Role of Nutrient Deprivation

Tumor cells prefer the utilization of ATP via aerobic glycolysis, which is known as the “Warburg effect” [73]. Compared to mitochondrial respiration, aerobic glycolysis provides survival advantages such as more rapid ATP synthesis and increased tolerance to fluctuating oxygen levels [73]. Tumor cells sustain anabolic processes using glucose and can adapt to increased levels of reactive oxygen species [74,75]. Moreover, hyperactive glycolysis inhibits mitochondrial respiration caused by enzymatic competition for the ADP cytoplasmic pool. Additionally, overexpression of hexokinase isoform II (HK-II) in cancer cells has been shown to associate with voltage-dependent anion channels (VDAC). It is crucial for neoplastic cell survival via regulation of pro-apoptotic factors such as: cytochrome c (cyt c), apoptosis-inducing factor (AIF) and Bcl-2-associated X protein (Bax) release [76].

Serum deprivation causes stress and induces cell death. A lack of nutrients can induce apoptosis, increased ROS levels and caspase activity. Cellular starvation decreases glycolytic metabolism ratio and stimulates oxidative phosphorylation. A high metabolic rate is specific for MPNST and indicates its worse outcome. MPNST has shown dependence on glycolysis as tumor cells generally prefer the glucose intake and therefore react with decreased viability under starvation conditions [77]. Novel antitumor therapies may include agents that affect tumor angiogenesis, drugs that decrease blood glucose levels, or novel therapeutic approaches to create a nutrient-deprivation environment [78].

Nutrient deprivation facilitates the therapeutic effect of some agents, e.g., 3-bromopyruvate (3-BrPA) in MPNST [77]. 3-BrPA influences the mitochondria functions in glioblastoma cells, elevates ROS synthesis and decreases the viability of tumor cells adapted to the increased ROS levels [79]. MPNST treatment with 3-BrPA resulted in a decrease of ROS production, which approximately resembled the cellular viability response; however, the proteasome activator 28 γ (PA28γ) overexpression was attenuated [77]. Cells with enhanced expression of PA28γ are resistant to apoptosis due to increased levels of anti-apoptotic B-cell lymphoma-extra-large protein (Bcl-xL) important for homeostasis of mitochondria [80]. The mechanisms of ROS generation via mitochondrial complexes I and III were affected by 3-BrPA at higher concentrations, although PA28γ overexpression leads to reduced sensitivity [77]. High expression of PA28γ has been reported in various cancer types and serves as a representative model, especially for MPNST. Inactivation of PA28γ and TP53 prevents cell death [81]. In MPNST, PA28γ overexpressing cells responded to treatment, but only at higher concentrations of 3-BrPA [77], probably due to additional genetic abnormalities other than p53 inactivation.

Increased glycolysis in cancer cells results in its conversion to lactate (instead of the translocation to the mitochondrial complex I) and thus the enhanced expression of lactate dehydrogenase (LDH) [82]. The regeneration of NAD+ to NADH during conversion of pyruvate into lactate allows to maintain stable glycolytic flux in cancer cells and is thought to prevent the activity of the mitochondrial complex I and an enhanced ROS synthesis [83]. Under normal conditions, PA28γ cells overexpressing showed a milder increase of L-lactate levels in response to low doses of 3-BrPA, but nutrient deprivation strongly sensitized PA28y cells to 3-BrPA and restrained lactate conversion [77]. These results suggest a potential role of PA28γ in lactate metabolism control via regulation of LDH activity and impact on 3-BrPA treatment that can be partially reversed by nutrient deficiency. Data indicate an important role of lactate in the tumor microenvironment, the interactive tumor and stromal cells crosstalk [84].

## 7. ER-Stress and Its Regulators

ER stress is induced when unfolded proteins accumulate in the endoplasmic reticulum due to various external and intracellular factors. One of the hallmarks of cancer cells is high levels of ER stress, caused by factors such as nutrient deprivation, oxidative stress and hypoxia followed by high mutational load and copy number variation [14]. Aneuploidy has recently been shown to induce previously described proteotoxic stress in both normal and cancer cells [19].

Neurofibromin encoded by NF-1, which negatively regulates Ras via Ras-GTP hydrolysis [85]. NF1-deficient tumors are driven by aberrant Ras activation [86]. In NF1-deficient tumors, mTOR has been shown to be hyperactivated due to aberrant Ras signaling. MPNSTs are highly aneuploid tumors with the constitutive activation of Ras and, therefore, might be subject to substantial ER stress. It has been shown that ER stress levels were significantly higher in tumors compared with normal peripheral nerves [43]. Moreover, incubation with agents that induce ER stress-triggered MPNST cell death at concentrations that did not affect the viability of normal cells [43]. Therefore, ER stress appears to be a promising strategy for MPNST treatment.

In an NF1/TP53-mutant MPNST murine model, mTOR inhibitors (mTORi) suppressed tumor growth in a potent, but cytostatic, manner and ultimately became resistant to treatment [87,88]. Therefore, the identification of alternative strategies in combination with mTORi may be beneficial. Oncogenic Ras also causes ER stress, and when the ER stress level becomes insurmountable, cell death ensues, suggesting factors that enhance ER stress may be developed as anticancer agents [89]. ER stress induction using HSP90 inhibitors coupled with mTORi led to a reduction in MPNST tumor size [43]. It has only been observed in tumors treated with the combination of both inhibitors but not in tumors exposed to either of the same agent.

Heat Shock Protein 90 maintains protein homeostasis through multiple actions: the proper folding, intracellular disposition and proteolytic turnover of many key regulators of cell growth, differentiation and survival [90]. HSP90 also directly stabilizes two ER stress-sensors: IRE1 and pPERK/PERK [91]. Therefore, HSP90 inhibitors can be expected to promote ER stress in cancer cells via two cooperating mechanisms: first, by directly impairing global protein folding disruption as well as by adaptive responses inactivation. Several ER stress inducers, including the HSP90 inhibitor IPI-504, have been found to cooperate with rapamycin in promoting dramatic tumor regression in two distinct Ras driven-cancers, including MPNST [43].

Another study by Kim et al. (2020) determined the safety, tolerability, and recommended dose of ganetespib (a small molecule inhibitor of HSP90) and sirolimus (mTOR inhibitor) in patients with unresectable/refractory MPNST [92]. The combination of HSP90 inhibitors to enhance endoplasmic reticulum stress with mTOR inhibition results in tumor shrinkage in a murine MPNST model. Unfortunately, the combination therapy was not successful, and the study did not meet parameters for further evaluation in MPNST [92].

Another way to induce ER stress in MPNST cells is to inhibit the activity of Ubiquitin Specific Peptidase 9 X-Linked (Usp9X). Usp9X is a deubiquitinating enzyme that plays a role in regulating protein degradation by modulating ubiquitin conjugation to its targets and thereby controlling their proteasomal turnover [93,94]. Usp9X plays a crucial role in various malignant tumors of the nervous system [95]. Additionally, Usp9X affects cell survival by regulating the levels of anti-apoptotic Bcl-2 family members, including myeloid cell leukemia 1 (Mcl-1), or by inhibiting apoptosis proteins and stabilizing the X-linked inhibitor of apoptosis protein (XIAP) and thus controlling the death control of mitotic cells [96]. Interference with Usp9X results in degradation of these anti-apoptotic proteins [94,96].

The regulatory mechanism by which Usp9X inhibition results in decreased viability of MPNST cells appears to be complex. Usp9X inhibition resulted in an ER stress response with increased ATF3 and ATF4, two markers of ER stress, followed by overexpression of pro-apoptotic Noxa and a subsequent decrease in Mcl-1 protein levels [97]. Significant reductions in Mcl-1 protein levels were also observed in ST88-14 xenografts undergoing treatment with WP1130.

Ultrastructural examination of MPNST cells following Usp9X interference or pharmacological inhibition with WP1130 treatment showed features of ER stress (cytoplasmic vacuolization and swelling of endoplasmic reticulum). Moreover, paraptosis, a type of caspase-independent cell death associated with ER damage, was also observed [18]. These results suggest a relation between Usp9X and ER stress in the context of MPNST. Additionally, an overexpression of Noxa, a pro-apoptotic factor regulated by ATF3 and ATF4 and preceded the Mcl-1down-regulation [98,99,100]. Noxa hyperactivation facilitates the release of BAK and BIM and triggers BAX/BAK mediated apoptosis.

## 8. Conclusions

Malignant peripheral nerve sheath tumors are the major cause of death among neurofibromatosis-1 patients and do not have a promising therapeutic strategy due to their abnormal cell metabolism and pathophysiology.

While proteotoxic and oxidative stress in MPNST cells can arise for distinct reasons, there is a crosstalk between cellular response pathways, linking the mitochondria and ER in a complex and interdependent manner. UPR induction causes an influx and efflux of calcium from the ER, which causes depolarization of mitochondrial membrane followed by increased ROS production, which in turn can cause protein folding deterioration [101].

On the other hand, oxidative stress affects the proper protein folding, which provokes UPR, leading to ER stress induction and further mitochondrial damage [102]. At moderate levels of stress the cell can evoke the balancing mechanisms such as UPR and the glutathione and thioredoxin systems capable of reducing the ER stress level and scavenging ROS. Moreover, if cellular stress level is unassailable and UPR-adaptive pathways do not function sufficiently, it will damage the ER and mitochondria, eventually leading to cell death [103].

The increased levels of oxidative and proteotoxic stress common to cancer cells make them more sensitive to various inducers or modulators of cellular stress responses. MPNST have high basal level of ER-stress, making them very sensitive to a number of ER-stress inducing agents (e.g., tunicamycin, thapsigargin, and HSP90 inhibitors), and such treatment induces apoptosis in tumor cells in vitro. However, as it was demonstrated in the murine model of MPNST, monotherapy with these agents did not reduce tumor size in mice [43]. Better effects are observed with ER stress inducing agents combined with the rapamycin (mTOR inhibitor) therapy. Rapamycin itself is a cytostatic agent, but together with ER stressors it induces cytotoxic response in MPNST cells [43,88]. This effect seems to be highly dependent on ROS presence (as a result of ER stress induction) and ascorbic acid, a ROS scavenger, could inhibit the cytotoxic effect [43]. 

Tunicamycin, thapsigargin, and HSP90 inhibitors used as monotherapy lead to the ROS synthesis and an elevated ER-stress level, but the MPNST cells are able to weaken this stress effect by UPR pathways activation. It has been also found that mTOR inhibitors restrain the activity of the pentose phosphate pathway and since the glutathione ROS scavenging system is highly dependent on NADPH to maintain a reduced GSH pool. When mTOR inhibitors are combined with ER stress inhibitors, the cell has reduced ability to attenuate the oxidative stress evoked ER stress inducers, what results in mitochondria and ER damage in MPNST cells [43,104]. The use of ER stress inducing agents and mTOR inhibitors seems to be the first type of chemotherapy to induce tumor shrinkage in aggressive mouse model of MPNST, making it the most promising therapeutic strategy. Among compounds that induce ER stress, only the most promising HSP90 inhibitors are clinically tested. A clinical trial of restapimycin (IPI-504) and everolimus (an mTOR inhibitor) has been conducted in patients with KRAS-mutant non-small cell lung cancer, however study results are not yet public (NCT01427946). A Phase I/II of second-generation HSP90 inhibitor ganetespib (STA-9090) and sirolimus, despite promising preclinical rationale and tolerance of the combination therapy, no response was observed, and the study did not meet parameters for further evaluation in MPNST [92]. 

Unfortunately, none of HSP90 inhibitors have shown satisfactory efficacy for FDA approval, and it is possible that a tolerable therapeutic window for this particular combination will not be identified. However, based on the proposed mechanism of stressors synergy, a large number of ER stress modulators or ROS modulators have the potential to induce the apoptosis in cancer cells in combination with mTOR or other pathways inhibitors.
